# Supplementary material for: Citrate supplement facilitated muscle growth and renal maturation
Source: PLoS One. 2026 Jan 20;21(1):e0339198. doi: 10.1371/journal.pone.0339198 (PMC12818636; doi:10.1371/journal.pone.0339198)
Supplement: Attachment — Submitted filename: review Plos.docx [file pone.0339198.s006.docx]

Authors report on the role of potassium/sodium citrate in muscle growth and renal maturation

Abstract

Introduction under abstract lacks clarity and sentences are disjointed

Method

Authors administered 2000 mg/kg body weight of PCSC by oral gavage to PCSC group daily for one week. What is the rationale for choosing 2000mg/kg/bw?

Why did the authors choose 8 days for the drug/agent administration?

Any reason why authors used only one dose level rather than 3-5 to examine dose-dependency as a proof of cause and effect?

Statistics

Authors calculated muscle growth with the formula: MG= (weight of 70 vastus lateralis muscle)/[(body weight on day 8)- (body weight on day 0)], kindly provide reference

Formula for determining the renal function should be provided

Results and discussion

Reference appropriate sections eg., A significant increase in malate levels was observed in the kidneys. Cytoplasmic citrate can be 136 converted to malate by malate enzymes 1 and 3, utilizing NADH as a cofactor. The increased 137 malate/aspartate ratio suggests activation of the malate-aspartate shuttle in renal mitochondria.
