# Supplementary material for: Citrate supplement facilitated muscle growth and renal maturation
Source: PLoS One. 2026 Jan 20;21(1):e0339198. doi: 10.1371/journal.pone.0339198 (PMC12818636; doi:10.1371/journal.pone.0339198)
Supplement: Attachment — Submitted filename: response.docx [file pone.0339198.s007.docx]

**Dear Editor,**

Thank you for giving me the opportunity to review the manuscript in ***PLOS ONE***

**Titled** Citrate supplement facilitated muscle growth and renal maturation. I appreciate your trust in my feedback, and I look forward to contributing to the review process.

**General comments:**

In my opinion, the current version of the manuscript appears limited in scope and depth to meet the standards typically required for journal publication. To strengthen the paper, I recommend expanding the literature review, providing more detailed methodology and analysis, and offering a deeper discussion of the results. Additionally, clarifying the research objectives and emphasizing the novelty and contribution of your work would significantly enhance the manuscript's impact and clarity.

**Specific comments:**

**Abstract**

- Please ensure that all abbreviations are spelled out in full the first time they appear in the abstract for clarity. This helps readers unfamiliar with the terms to better understand your work
- The abstract/background section appears limited in scope and depth. I recommend expanding it to provide a clearer context for the study, including key motivations, and the specific research gap being addressed. This will help readers better understand the significance and novelty of your work from the outset.

**Introduction**

- The introduction is currently too brief and does not sufficiently establish the physiological or mechanistic link between renal dysfunction and muscle fatigue. To improve the manuscript, I recommend expanding the introduction to include:
- (1) the role of kidney function in metabolic homeostasis,
- (2) how impaired renal function can lead to systemic metabolic disturbances
- (3) the downstream effects of these disturbances on skeletal muscle metabolism and fatigue.

(decreased creatinine and NADPH/NADP⁺ ratio may reflect altered energy metabolism and oxidative stress in muscle tissue, while changes in urea and amino acid metabolism (e.g., N,N-dimethylglycine, sarcosine) in kidney tissue could signal impaired nitrogen handling and mitochondrial dysfunction, both of which are known to contribute to fatigue)

**material and methods**

**Animals and experimental protocol**

- reference for 2000 mg/kg body weight of PCSC

**Extraction of metabolites and preparations for metabolome analyses**

- The section on “Extraction of metabolites and preparations for metabolome analyses” requires additional methodological detail to ensure reproducibility and transparency. The Method section should be expanded to include detailed information on animal handling and authentication.

**Results**

- I noticed that the pathology figure, which provides valuable visual evidence supporting the study’s findings, is currently included only in the supplementary materials. I strongly recommend moving this figure into the main manuscript. Specifically, it would be appropriate to reference and describe it within both the Methodology and Results sections as separate paragraphs.

**Discussion**

- Please enrich the Discussion section by adding references to previous studies on octreotide and deferoxamine to better support and contextualize your findings. Including relevant literature will help demonstrate how your results align with or differ from prior work, highlighting the novelty and significance of your study.
